# Kidney Transplant Recipient Attitudes Toward a SARS-CoV-2 Vaccine

**DOI:** 10.1097/TXD.0000000000001171

**Published:** 2021-06-10

**Authors:** Michael T. Ou, Brian J. Boyarsky, Laura B. Zeiser, Teresa Po-Yu Chiang, Jake Ruddy, Sarah E. Van Pilsum Rasmussen, Jennifer Martin, Jennifer St. Clair Russell, Christine M. Durand, Robin K. Avery, William A. Werbel, Matthew Cooper, Allan B. Massie, Dorry L. Segev, Jacqueline M. Garonzik-Wang

**Affiliations:** 1 Department of Surgery, Johns Hopkins University School of Medicine, Baltimore, MD.; 2 National Kidney Foundation, New York, NY.; 3 Department of Medicine, Johns Hopkins University School of Medicine, Baltimore, MD.; 4 Department of Epidemiology, Johns Hopkins School of Public Health, Baltimore, MD.; 5 Medstar Georgetown Transplant Institute, Washington, DC.; 6 Scientific Registry of Transplant Recipients, Minneapolis, MN.

## Abstract

A widely accepted severe acute respiratory syndrome 2 (SARS-CoV-2) vaccine could protect vulnerable populations, but the willingness of solid organ transplant recipients (SOTRs) to accept a potential vaccine remains unknown.

**Methods.:**

We conducted a national survey of 1308 SOTRs and 1617 non-SOTRs between November 11 and December 2, 2020 through the network of the National Kidney Foundation.

**Results.:**

Respondents were largely White (73.2%), female (61.1%), and college graduates (56.2%). Among SOTRs, half (49.5%) were unsure or would be unwilling to receive a SARS-CoV-2 vaccine once available. Major concerns included potential side effects (85.2%), lack of rigor in the testing and development process (69.7%), and fear of incompatibility with organ transplants (75.4%). Even after the announcement of the high efficacy of the mRNA-1273 vaccine (Moderna Inc.) at the time of survey distribution, likeliness to receive a vaccine only slightly increased (53.5% before announcement versus 57.8% after the announcement). However, 86.8% of SOTRs would accept a vaccine if recommended by a transplant provider.

**Conclusions.:**

SOTRs reported skepticism in receiving a potential SARS-CoV-2 vaccine, even after announcements of high vaccine efficacy. Reassuringly, transplant providers may be the defining influence in vaccine acceptance and will likely have a critical role to play in promoting vaccine adherence.

## INTRODUCTION

A widely-accepted severe acute respiratory syndrome 2 (SARS-CoV-2) vaccine could protect the community and vulnerable populations. However, the safety and efficacy of SARS-CoV-2 vaccines in solid organ transplant recipients (SOTRs) are currently unknown as clinical trials have excluded immunocompromised individuals.^1–5^ Furthermore, although mRNA vaccine platforms offer greater flexibility in antigen manipulation and increased speed of development,^6^ this relatively new platform has also not been tested in SOTRs.^1,7^ Coupled with the lack of long-term data, concerns over safety may impact the willingness of SOTRs to accept an SARS-CoV-2 vaccine once individually available.

Although vaccines provide direct immunity to those vaccinated, they also provide indirect protection to unvaccinated individuals via herd immunity. One study estimated that at least two-thirds of the population need to be vaccinated before coronavirus disease 2019 (COVID-19) herd immunity develops,^8^ yet projected global acceptance of SARS-CoV-2 vaccine dips to only half of the population in some countries,^9^ and surveys of projected acceptance in the United States within non-SOTR populations vary widely.^10–13^ Despite this heterogeneity in vaccine acceptance patterns, physician recommendation may play an important role in vaccine acceptance, often being cited as the top reason for vaccination.^14–17^ For SOTRs, transplant provider recommendation may play a particularly critical role in vaccine acceptance, given the close relationship and trust SOTRs have with their transplant team.^18,19^ However, it is unknown whether SOTRs or their household contacts will widely accept a vaccine if a provider recommendation can overcome vaccine skepticism, or whether announcements of high efficacy in vaccine trials have changed vaccine perceptions.

To elicit the attitudes toward a potential SARS-CoV-2 vaccine in SOTRs across the United States, we conducted a national survey of SOTRs and non-SOTRs between November 11 and December 2, 2020. We gathered data in 2 major domains: (1) perceptions of a SARS-CoV-2 vaccine and (2) the impact of the pandemic on daily life and mental health. To assist future vaccine uptake efforts, we also identified potential barriers to vaccination. Understanding the perceptions of SOTRs will be a fundamental step in addressing vaccine concerns and promoting widespread acceptance in this vulnerable population.

## MATERIALS AND METHODS

### Participants and Survey Distribution

We distributed the survey through the National Kidney Foundation’s (NKF) network of 2806 SOTRs and 12 476 non-SOTRs (including family members, spouses, caregivers, and living donors) via email. A link to the survey was also posted twice to the NKF’s social media platforms and was clicked a total of 364 times. For the purposes of calculating a response rate, we include the 364 social medial post views as unique survey invitations (and thus were included in our source population). Unfortunately, we were unable to determine if the social media post viewers were SOTRs or non-SOTRs. Thus, we report a range of response rates for each group, under the assumption that all or none were SOTRs. Thus, our response rate for SOTRs was 41.2%–46.6% and our response rate for non-SOTRs was 12.6%–13.0%. The overall response rate was 18.7%. The survey was conducted between November 11 and December 2, 2020, deemed an exempt research study by the institutional review board at Johns Hopkins School of Medicine (IRB00266679) and was hosted by Qualtrics (Provo, UT).

### Survey Design

Questions were developed through an iterative process, based on a thorough review of the literature and discussion with 3 transplant surgeons, 3 transplant infectious diseases physicians, and a survey research expert. The input was also obtained from other members of the transplant team and the NKF. The survey and was piloted in a cohort of 11 transplant recipients, family members, and patients with kidney disease and found to function as intended.

### Survey Domains

The survey contained a total of 42 questions. Participant demographics including sex, race, ethnicity, education, household income, household size, marital status, employment status, and location were collected. Medical and transplant history was elicited, as well as test status, hospitalizations, and medical interventions related to COVID-19 infection. We studied 2 major domains: perceptions of a SARS-CoV-2 vaccine and the impact of the pandemic on daily life and mental health. The first domain included questions about willingness to obtain a potential SARS-CoV-2 vaccine, the recommendation to others, and perceptions about vaccine use in transplant recipients. Specific factors that influence vaccine hesitancy were also determined, based on open-ended themes previously identified in a survey of US adults.^11^ The second domain focused on understanding the impact of the COVID-19 pandemic on daily routine, access to medical care, and life circumstances such as job and income stability. This domain also included the Coronavirus Anxiety Scale to identify probable cases of dysfunctional anxiety associated with the COVID-19 crisis (Table S1, SDC, http://links.lww.com/TXD/A337).^20,21^ The survey took, on average, <10 min.

### Statistical Analysis

All statistical analyses were performed using Stata 16.1 for Windows (College Station, TX). We tested the association between vaccination attitudes and binary variables by Fisher’s exact test, and continuous variables using the Wilcoxon rank-sum test. We reported *P* values with an α of 0.05 for statistical significance.

We compared SOTRs to all non-SOTRs respondents. Among the non-SOTR respondents, we also compared those who lived in the same house to an SOTR (household contact) to those who did not (nonhousehold contacts). Although our survey was distributed after the announcement of high efficacy in the BNT162b1 vaccine (Pfizer Inc.), announcement of 94.5% efficacy in the mRNA-1273 vaccine (Moderna Inc.) occurred on November 16, 2020. We compared vaccine attitudes before and after this announcement by stratifying between those that answered before and after November 17, 2020. This date was used to account for the lag between news availability and awareness by the general public.

Among SOTRs who did not know that they were being excluded from vaccine clinical trials, we compared their likelihood of vaccination before and after learning this information. We used a 5-point Likert scale when asking about the likelihood of receiving a potential vaccine but collapsed responses into binary variables; those extremely likely or likely we included in one group whereas those who were unsure, unlikely, or extremely unlikely into another.

The Coronavirus Anxiety Scale, which has a 90% sensitivity and 85% specificity at a cutoff score of 9, was originally validated in a population with known COVID-19 anxiety. Repeat measurements found that a cutoff score of 5 may be more appropriate in populations in which the proportion of COVID-19 anxiety is unknown.^20^ We included both for comprehensiveness.

## RESULTS

### Study Population

A total of 2925 people responded with approximately 44.7% being SOTRs. The majority of respondents were White (73.2%) and female (61.1%), with a wide distribution of age, geographic region, and household income (Table 1). A large number had at least a college degree (56.2%) and were married (56.1%). Seventy-seven respondents (2.6%) had tested positive for COVID-19 and among those positive, 18 respondents (22.2%) had been hospitalized. The majority (98.9%) of SOTRs were kidney transplant recipients. Compared to non-SOTRs, more SOTRs were male (37.9% versus 29.9%, *P* < 0.001), had at least a college degree (59.0% versus 54.0%, *P* = 0.01), were employed (41.9% versus 36.2%, *P* < 0.01), and believed they would get seriously ill from COVID-19 in the next 6 mo (14.6% versus 9.8%, *P* < 0.001). Less SOTRs intended to receive a vaccine compared to non-SOTRs (50.5% versus 60.7%, *P* < 0.001).

**TABLE 1.** Participant characteristics, by transplant status

Factor	Total	SOTRs	Non-SOTRs	*P*
N	2925	1308 (44.7%)	1617 (55.3%)	
Age category				
18–29	102 (3.5%)	51 (3.9%)	51 (3.2%)	<0.001
30–49	684 (23.4%)	342 (26.1%)	342 (21.2%)	
50–64	1022 (34.9%)	503 (38.5%)	519 (32.1%)	
>64	968 (33.1%)	346 (26.5%)	622 (38.5%)	
Not reported	149 (5.1%)	66 (5.0%)	83 (5.1%)	
Gender				
Female	1786 (61.1%)	744 (56.9%)	1042 (64.4%)	<0.001
Male	980 (33.5%)	496 (37.9%)	484 (29.9%)	
Others	2 (0.1%)	1 (0.1%)	1 (0.1%)	
Not reported	157 (5.4%)	67 (5.1%)	90 (5.6%)	
Race				
White	2142 (73.2%)	955 (73.0%)	1187 (73.4%)	0.1
Black	251 (8.6%)	115 (8.8%)	136 (8.4%)	
Asian	98 (3.4%)	54 (4.1%)	44 (2.7%)	
AI/AN/Pacific Islander	38 (1.3%)	11 (0.8%)	27 (1.7%)	
Multiracial	73 (2.5%)	33 (2.5%)	40 (2.5%)	
Other	98 (3.4%)	39 (3.0%)	59 (3.6%)	
Not reported	225 (7.7%)	101 (7.7%)	124 (7.7%)	
Hispanic ethnicity				
Non-Hispanic	2389 (81.7%)	1090 (83.3%)	1299 (80.3%)	0.5
Hispanic	202 (6.9%)	87 (6.7%)	115 (7.1%)	
Not reported	334 (11.4%)	131 (10.0%)	203 (12.6%)	
US geographic region				
Northeast	522 (17.8%)	251 (19.2%)	271 (16.8%)	0.02
Midwest	698 (23.9%)	334 (25.5%)	364 (22.5%)	
South	881 (30.1%)	374 (28.6%)	507 (31.4%)	
West	548 (18.7%)	236 (18.0%)	312 (19.3%)	
Non-US residence	116 (4.0%)	41 (3.1%)	75 (4.6%)	
Not reported	160 (5.5%)	72 (5.5%)	88 (5.4%)	
Educational attainment				
High school diploma or less	357 (12.2%)	157 (12.0%)	200 (12.4%)	0.01
Some college	724 (24.8%)	290 (22.2%)	434 (26.8%)	
College graduate or more	1645 (56.2%)	772 (59.0%)	873 (54.0%)	
Not reported	199 (6.8%)	89 (6.8%)	110 (6.8%)	
Marital status				
Not married	1043 (35.7%)	444 (33.9%)	599 (37.0%)	0.06
Married	1642 (56.1%)	761 (58.2%)	881 (54.5%)	
Not reported	240 (8.2%)	103 (7.9%)	137 (8.5%)	
Employment status				
Not employed	1543 (52.8%)	651 (49.8%)	892 (55.2%)	<0.01
Employed	1134 (38.8%)	548 (41.9%)	586 (36.2%)	
Not reported	248 (8.5%)	109 (8.3%)	139 (8.6%)	
Annual household income (thousands)				
<30	490 (16.8%)	180 (13.8%)	310 (19.2%)	<0.001
30–59	557 (19.0%)	241 (18.4%)	316 (19.5%)	
60–100	597 (20.4%)	281 (21.5%)	316 (19.5%)	
≥100	630 (21.5%)	325 (24.8%)	305 (18.9%)	
Not reported	651 (22.3%)	281 (21.5%)	370 (22.9%)	
Household size				
1	454 (15.5%)	185 (14.1%)	269 (16.6%)	<0.001
2	1831 (62.6%)	868 (66.4%)	963 (59.6%)	
3 or more	639 (21.8%)	255 (19.5%)	384 (23.7%)	
Not reported	1 (<1%)	0 (0.0%)	1 (0.1%)	
Smoking status				
Never smoked	1871 (64.0%)	903 (69.0%)	968 (59.9%)	<0.001
Smoke currently or in the past	1050 (35.9%)	404 (30.9%)	646 (40.0%)	
Not reported	4 (0.1%)	1 (0.1%)	3 (0.2%)	
Received a flu vaccine in 2019 (n = 2804)				
No	451 (16.1%)	135 (10.8%)	316 (20.3%)	<0.001
Yes	2353 (83.9%)	1115 (89.2%)	1238 (79.7%)	
Have or will receive a flu vaccine in 2020 (n = 2798)				
No	363 (13.0%)	131 (10.5%)	232 (15.0%)	<0.001
Yes	2435 (87.0%)	1122 (89.5%)	1313 (85.0%)	
COVID-19 testing status (n = 2918)				
Tested negative	1223 (41.9%)	568 (43.6%)	655 (40.5%)	0.1
Tested positive	77 (2.6%)	39 (3.0%)	38 (2.4%)	
Inconclusive/never tested^*a*^	1618 (55.4%)	695 (53.4%)	923 (57.1%)	
Admitted to the hospital because of COVID-19 (n = 81)^*b*^				
No	63 (78%)	27 (69%)	36 (86%)	0.1
Yes	18 (22%)	12 (31%)	6 (14%)	
Perception of COVID-19 risk in next 6 mo (n = 2791)				
I don’t think I will get COVID-19	2146 (76.9%)	957 (76.9%)	1189 (76.9%)	<0.001
I think I will get a mild case of COVID-19	311 (11.1%)	106 (8.5%)	205 (13.3%)	
I think I will get seriously ill from COVID-19	334 (12.0%)	182 (14.6%)	152 (9.8%)	
Belief that SOTRs are more likely to have a severe course of COVID-19 (n = 2925)				
No	674 (23.0%)	445 (27.5%)	229 (17.5%)	<0.001
Yes	2251 (77.0%)	1079 (82.5%)	1172 (72.5%)	
Belief that SOTRs may get seriously ill from a COVID-19 vaccine (n = 2369)				
No	1190 (50.2%)	663 (51.6%)	527 (48.6%)	0.1
Yes	1179 (49.8%)	558 (51.4%)	621 (48.4%)	
Belief that COVID-19 vaccine may lead to an organ transplant rejection (n = 2111)				
No	1581 (74.9%)	883 (76.6%)	698 (72.8%)	0.04
Yes	530 (25.1%)	261 (27.2%)	269 (23.4%)	
Believe that COVID-19 vaccine would be safe for SOTRs (n = 2134)				
No	1288 (60.4%)	704 (60.1%)	584 (60.6%)	0.8
Yes	846 (39.6%)	379 (39.4%)	467 (39.9%)	
COVID-19 anxiety score, (n = 2813)				
Median (IQR)	0 (0–2)	0 (0–1)	0 (0–2)	0.1
Score ≥ 5	228 (8.1%)	102 (8.1%)	126 (8.1%)	>0.9
Score ≥ 9	57 (2.0%)	24 (1.9%)	33 (2.1%)	0.8
Intend to receive a COVID-19 vaccine (n = 2925)				
No	1283 (43.9%)	647 (49.5%)	636 (39.3%)	<0.001
Yes	1642 (56.1%)	661 (50.5%)	981 (60.7%)	
Organ transplanted (n = 1308)				
Kidney	–	1293 (98.9%)	–	–
Other	–	70 (1.1%)	–	–
Effect of knowing transplant recipient on decision to receive a vaccine^*c*^ (n = 606)				
No effect	–	–	349 (57.6%)	–
Less likely to receive vaccinate	–	–	25 (4.1%)	–
More likely to receive vaccine	–	–	232 (38.3%)	–

^*a*^Four survey participants had inconclusive COVID-19 test results.

^*b*^Responses collected from participants who had positive or inconclusive COVID-19 test results.

^*c*^Responses collected from non-SOTRs who indicated knowing an SOTR.

COVID-19, coronavirus disease 2019; SOTR, solid organ transplant recipient.

### Characteristics of SOTRs Unlikely to Accept a Vaccine

Six-hundred forty-seven (49.5%) SOTRs would be either unsure or unwilling to receive a SARS-CoV-2 vaccine once individually available (Table 2). Females (55.4%, *P* < 0.001), Blacks (71.3%, *P* < 0.001), and those of lower household income (62.8%, *P* < 0.001) and lower educational attainment (56.7%, *P* < 0.001) were more likely to be unsure or unwilling to receive a potential vaccine. Among SOTRs who did not receive a flu vaccine in 2019, 71.9% were unsure or would be unlikely to receive a potential SARS-CoV-2 vaccine compared to 28.1% who would be likely (*P* < 0.001). Among SOTRs who believed they would get seriously ill from COVID-19 in the next 6 mo, 48.9% indicated uncertainty or refusal of a potential vaccine.

**TABLE 2.** SOTR characteristics, stratified by intent to be vaccinated

Characteristic	Total	Likely to receive vaccine	Unsure/unlikely to receive vaccine	*P*
N	1308	661 (50.5%)	647 (49.5%)	
Age category				
18–29	51	33 (64.7%)	18 (35.3%)	<0.001
30–49	342	151 (44.2%)	191 (55.8%)	
50–64	503	251 (49.9%)	252 (50.1%)	
>64	346	204 (59%)	142 (41.0%)	
Gender				
Female	744	332 (44.6%)	412 (55.4%)	<0.001
Male	496	310 (62.5%)	186 (37.5%)	
Others	1	0 (0%)	1 (100.0%)	
Race				
White	955	534 (55.9%)	421 (44.1%)	<0.001
Black	115	33 (28.7%)	82 (71.3%)	
Asian	54	25 (46.3%)	29 (53.7%)	
AI/AN/Pacific Islander	11	4 (36.4%)	7 (63.6%)	
Multiracial	33	17 (51.5%)	16 (48.5%)	
Other	39	16 (41%)	23 (59.0%)	
Hispanic ethnicity				
Non-Hispanic	1090	578 (53%)	512 (47%)	0.4
Hispanic	87	42 (48.3%)	45 (51.7%)	
US geographic region				
Northeast	251	124 (49.4%)	127 (50.6%)	0.4
Midwest	334	173 (51.8%)	161 (48.2%)	
South	374	201 (53.7%)	173 (46.3%)	
West	236	124 (52.5%)	112 (47.5%)	
Non-US residence	41	16 (39%)	25 (61%)	
Educational attainment				
High school diploma or less	157	68 (43.3%)	89 (56.7%)	<0.001
Some college	290	131 (45.2%)	159 (54.8%)	
College graduate or more	772	434 (56.2%)	338 (43.8%)	
Marital status				
Married	761	207 (46.6%)	342 (44.9%)	<0.01
Not married	444	419 (55.1%)	237 (53.4%)	
Employment status				
Not employed	651	328 (50.4%)	323 (49.6%)	0.2
Employed	548	298 (54.4%)	250 (45.6%)	
Annual household income (thousands)				
<30	180	67 (37.2%)	113 (62.8%)	<0.001
30–59	241	109 (45.2%)	132 (54.8%)	
60–100	281	151 (53.7%)	130 (46.3%)	
≥100	325	224 (68.9%)	101 (31.1%)	
Household size				
1	185	91 (49.2%)	94 (50.8%)	0.08
2	868	456 (52.5%)	412 (47.5%)	
3 or more	255	114 (44.7%)	141 (55.3%)	
Smoking status				
Never smoked	903	461 (51.1%)	442 (48.9%)	0.6
Smoke currently or in the past	404	200 (49.5%)	204 (50.5%)	
Received a flu vaccine in 2019				
No	135	97 (71.9%)	38 (28.1%)	<0.001
Yes	1115	599 (53.7%)	516 (46.3%)	
Have or will receive a flu vaccine in 2020				
No	131	99 (75.6%)	32 (24.4%)	<0.001
Yes	1122	605 (53.9%)	517 (46.1%)	
Perception of COVID-19 risk in next 6 mo				
I don’t think I will get COVID-19	957	487 (50.9%)	470 (49.1%)	0.7
I think I will get a mild case of COVID-19	106	49 (46.2%)	57 (53.8%)	
I think I will get seriously ill from COVID-19	182	93 (51.1%)	89 (48.9%)	
Belief that transplant recipients are more likely to have a severe course of COVID-19				
No	229	137 (59.8%)	92 (40.2%)	<0.001
Yes	1079	569 (52.7%)	510 (47.3%)	
Belief that transplant recipients may get seriously ill from a COVID-19 vaccine				
No	527	168 (31.9%)	359 (68.1%)	<0.001
Yes	558	200 (35.8%)	358 (64.2%)	
Belief that a COVID-19 vaccine may lead to an organ transplant rejection				
No	698	239 (34.2%)	459 (65.8%)	<0.001
Yes	261	46 (17.6%)	215 (82.4%)	
Belief that a COVID-19 vaccine would be safe for transplant recipients				
No	584	401 (68.7%)	183 (31.3%)	<0.001
Yes	379	342 (90.2%)	37 (9.8%)	
Coronavirus Anxiety Scale				
Score ≥ 5	102	48 (47.1%)	54 (52.9%)	0.4
Score ≥ 9	24	11 (45.8%)	13 (54.2%)	0.7

COVID-19, coronavirus disease 2019; SOTR, solid organ transplant recipient.

### Barriers to Vaccine Acceptance

In respondents who indicated they would be uncertain or refuse an SARS-CoV-2 vaccine, side effects (85.2% SOTRs, 85.4% non-SOTRs), lack of rigor in testing and development (69.7% SOTRS, 69.5% non-SOTRS), and low efficacy (54.8% SOTRs, 56.0%) were cited as major concerns (Table 3). Compared to non-SOTRs, SOTRs were more likely to want information regarding their health compatibility with a potential vaccine (75.4% versus 59.0%, *P* < 0.001) and a recommendation by their physician (80.0% versus 57.9%, *P* < 0.001). Fewer SOTRs believed that vaccines “don’t work” (1.6% versus 3.7%, *P* = 0.02) and they had less concern about cost (23.6% versus 29.8%, *P* = 0.02), less mistrust of vaccines in general (7.7% versus 15.6%, *P* < 0.001), as well as less mistrust of pharmaceutical companies (29.4% versus 39.0%, *P* < 0.001).

**TABLE 3.** Barriers to vaccine acceptance

Barrier category and subcategory	SOTRs	Non-SOTRs	*P*
Participants unsure/unlikely to vaccinate	647	636	
Specific concerns about vaccine (n = 1234)			
Side effects	537 (85.2%)	516 (85.4%)	>0.9
Lack of rigor of testing	439 (69.7%)	420 (69.5%)	>0.9
Not wanting to be first to be vaccinated	378 (60.0%)	327 (54.1%)	0.04
Vaccine contents	364 (57.8%)	394 (65.2%)	<0.01
Efficacy	345 (54.8%)	338 (56.0%)	0.7
Need more information (n = 1256)			
Recommendation from doctor	511 (80.0%)	357 (57.9%)	<0.001
Personal health/transplant compatibility	482 (75.4%)	364 (59.0%)	<0.001
Personal immunity	188 (29.4%)	194 (31.4%)	0.5
Timing regarding state of pandemic	174 (27.2%)	172 (27.9%)	0.8
Cost	151 (23.6%)	184 (29.8%)	0.02
Antivaccine beliefs, attitudes, emotions (n = 1244)			
Others should get it first	204 (32.5%)	177 (28.7%)	0.2
Uncomfortable with vaccines	90 (14.3%)	124 (20.1%)	<0.01
Fear of vaccines	33 (5.3%)	54 (8.8%)	0.02
Don’t need any vaccine	28 (4.5%)	72 (11.7%)	<0.001
Don’t believe vaccine will work due to bad vaccine experiences	27 (4.3%)	44 (7.1%)	0.04
Religious beliefs	11 (1.8%)	15 (2.4%)	0.4
Vaccines don’t work in general	10 (1.6%)	23 (3.7%)	0.02
Don’t need because already infected	8 (1.3%)	15 (2.4%)	0.14
Doubts or mistrust (n = 1232)			
Vaccine development process	298 (47.9%)	310 (50.8%)	0.3
The government	221 (35.5%)	231 (37.9%)	0.4
Pharmaceutical companies	183 (29.4%)	238 (39.0%)	<0.001
CDC	105 (16.9%)	131 (21.5%)	0.04
Vaccines in general	48 (7.7%)	95 (15.6%)	<0.001
Participants likely to vaccinate	661	981	
Reasons less likely to vaccinate (n = 1630)			
Make me ill	275 (42.0%)	307 (31.5%)	<0.001
Paying out of pocket	114 (17.4%)	223 (22.8%)	<0.01
Minor side effects	80 (12.2%)	101 (10.3%)	0.3
Travel distance too far	66 (10.1%)	182 (18.6%)	<0.001
COVID-19 cases fall to 0	56 (8.6%)	118 (12.1%)	0.03
Already infected	29 (4.4%)	59 (6.0%)	0.2
Need to obtain vaccine more than once	25 (3.8%)	59 (6.0%)	0.052
Can’t get time off work	5 (0.8%)	19 (1.9%)	0.06

^*a*^In each category, n refers to the number of survey participants who provided a response.

CDC, Center for Disease Control and Prevention; COVID-19, coronavirus disease 2019; SOTR, solid organ transplant recipient.

**FIGURE 1.**
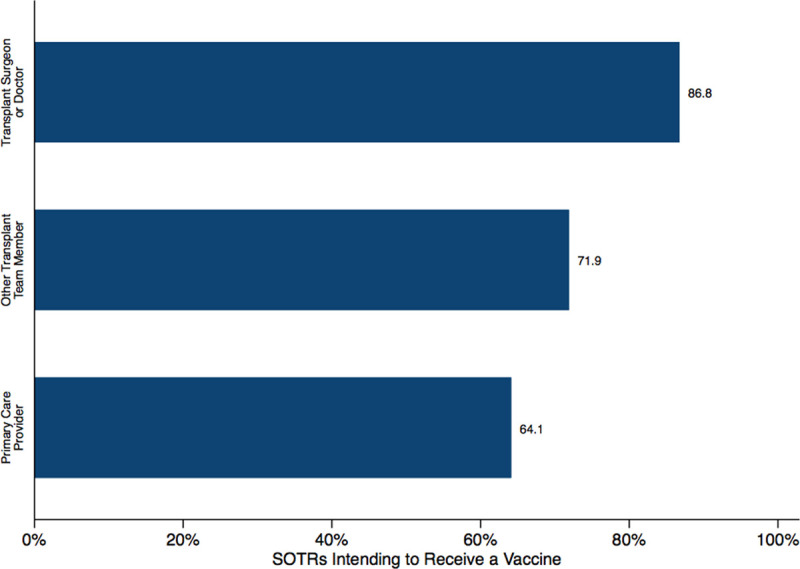
SOTRs’ intent to receive a vaccine based on recommendations from type of healthcare provider. SOTR, solid organ transplant recipient.

**FIGURE 2.**
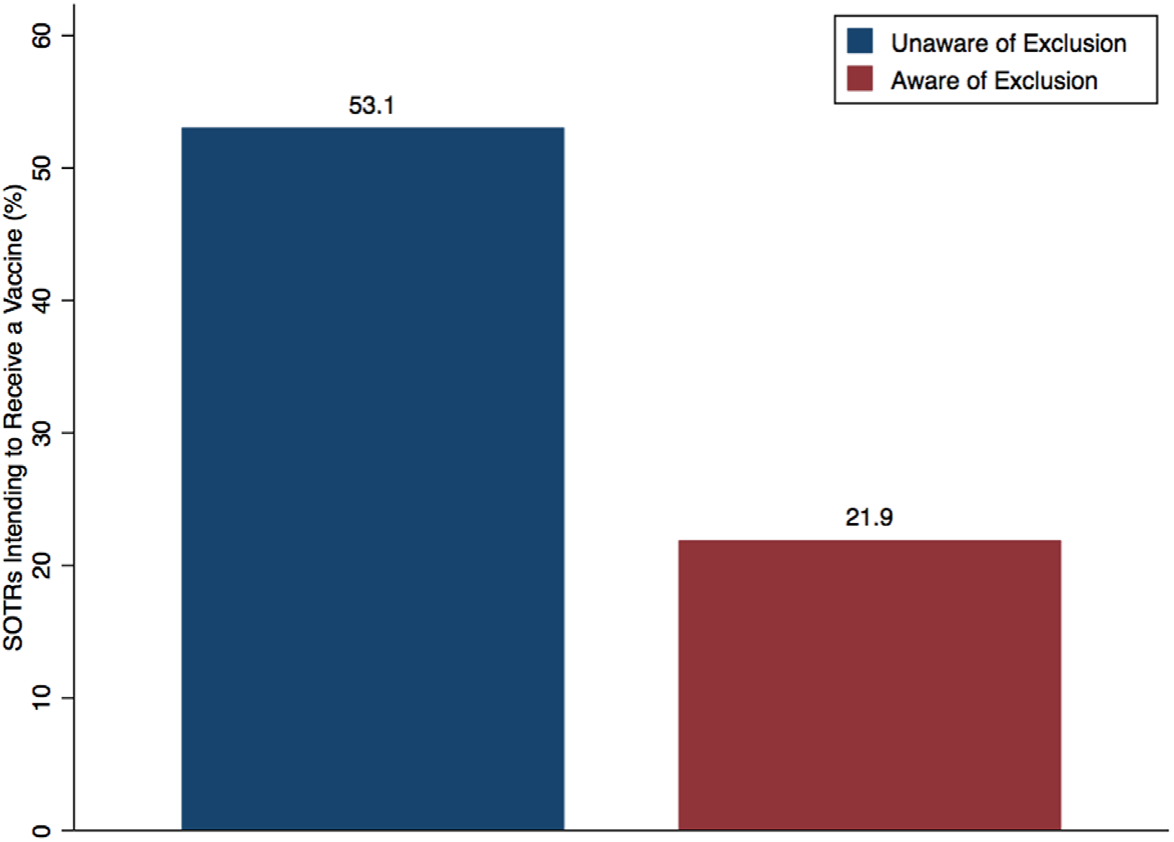
SOTRs’ intent to receive a vaccine before and after being made aware that SOTRs were excluded from clinical trials of COVID-19 vaccines. n = 982 (75.1%) respondents who were unaware that clinical trials excluded SOTRs. COVID-19, coronavirus disease 2019; SOTR, solid organ transplant recipient.

Among respondents who would be likely to accept vaccination, we asked what barriers would decrease vaccine adherence. Compared to non-SOTRs, SOTRs were less likely to indicate cost (17.4% versus 22.8%, *P* < 0.01) and travel distance (10.1% versus 18.6%, *P* < 0.001) as reasons that would prevent them from receiving a vaccine. Roughly two-thirds (61.6%) of people responded to the survey after the major announcement about vaccine efficacy in mRNA-1273 vaccine (Moderna Inc.) and respondents were only slightly more likely to agree to receive a vaccine (57.8% after announcement versus 53.5% before announcement) (Table S2, SDC, http://links.lww.com/TXD/A337). However, there were no significant differences in specific concerns about the vaccine itself. Although only half of SOTRs would be likely to receive a vaccine, 86.8% of SOTRs would receive a vaccine if recommended by a transplant surgeon or doctor, 71.9% if the recommendation came from a nonphysician transplant team member (ex. nurse, coordinator, physician assistant, etc), and 64.1% if from a primary care provider (*P* < 0.001) (Figure 1). Of the SOTRs, 75.1% did not know that SOTRs were being excluded from phase III trials, and among them, 21.9% would be likely to receive a vaccine compared to the 50.5% who would be likely before they were informed of this exclusion (Figure 2).

### Attitudes of Household Contacts

**FIGURE 3.**
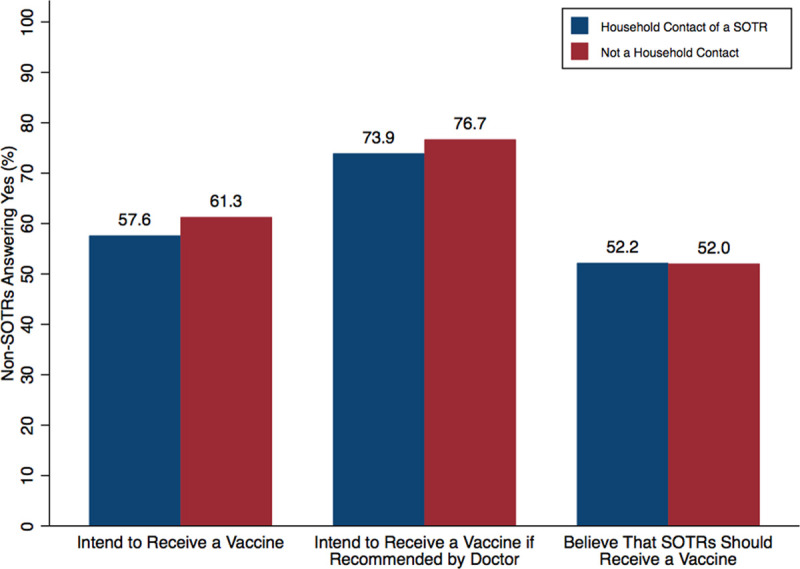
Vaccine attitudes in non-SOTRs, based on status of household contact of an SOTR. Of non-SOTRs, 606 (37.5%) of respondents lived with a SOTR (household contact). SOTR, solid organ transplant recipient.

**FIGURE 4.**
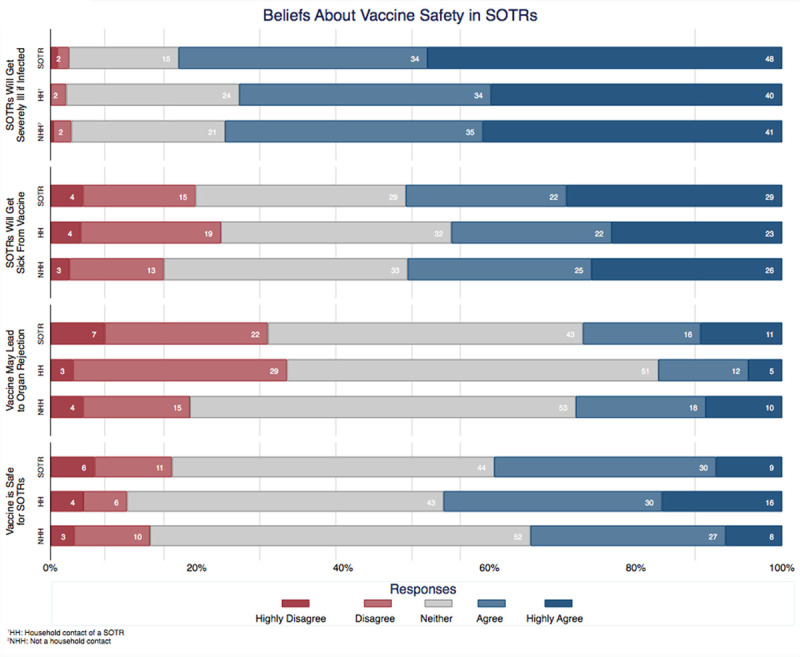
Beliefs about vaccine safety. SOTR, solid organ transplant recipient.

Of non-SOTRs, 606 (37.5%) respondents lived with an SOTR. Only 57.6% of these household contacts intend to receive a vaccine, a similar number (61.3%) to non-SOTR respondents who did not live with a SOTR (Figure 3). If recommended by a physician, 73.9% of household contacts would agree to receive a vaccine, whereas only 52.2% believed SOTRs should be vaccinated. Compared to non-SOTRs respondents who are not household contact, a larger proportion of household members believed that a vaccine would be safe for SOTRs (46% versus 35%) (Figure 4).

### Impact on Daily Life and Mental Health

Compared to non-SOTRs, SOTRs were more likely to be practicing social distancing (92.3% versus 89.7%, *P* = 0.02) and self-quarantine (12.3% versus 7.6%, *P* < 0.001) (Table 4). Non-SOTRs had more difficulty paying for basic necessities (43.4% versus 36.0%, *P* = 0.04), affording medical care (16.4% versus 10.8%, *P* = 0.02), and accessing a device for virtual healthcare (17.4% versus 9.8%, *P* < 0.01). However, severe anxiety related to the COVID-19 pandemic was low in both SOTRs and non-SOTRs (1.9% versus 2.1%, *P* = 0.8).

**TABLE 4.** COVID-19 impact, by transplant status

Impact Category and Subcategory	SOTRs	Non-SOTRs	*P*
N	1308 (44.7%)	1617 (55.3%)	
Daily activities			
Wearing a mask in public	1180 (95.2%)	1447 (94.3%)	0.3
Practicing social distancing	1145 (92.3%)	1377 (89.7%)	0.02
Staying home as much as possible	1097 (88.5%)	1353 (88.1%)	0.8
Not leaving home at all (in quarantine)	152 (12.3%)	116 (7.6%)	<0.001
Life circumstances			
Working fewer h	166 (47.8%)	196 (41.3%)	0.07
Loss of job	108 (31.1%)	140 (29.5%)	0.6
Loss of financial support, not job related	74 (21.3%)	107 (22.5%)	0.7
Loss of childcare	23 (6.6%)	45 (9.5%)	0.2
Loss of housing	5 (1.4%)	14 (2.9%)	0.2
Access to medical care			
Difficulty attending healthcare appointments	338 (84.9%)	425 (84.0%)	0.7
Difficulty paying for basic items (eg, food and clothing)	125 (36.0%)	206 (43.4%)	0.04
Difficulty obtaining medications	84 (21.1%)	111 (21.9%)	0.8
Difficulty affording medical care	43 (10.8%)	83 (16.4%)	0.02
Difficulty accessing a device for virtual healthcare	39 (9.8%)	88 (17.4%)	<0.01
Loss of health insurance	21 (6.1%)	27 (5.7%)	0.9
Gain of health insurance (eg, Medicaid)	9 (2.6%)	22 (4.6%)	0.1
Coronavirus Anxiety Scale			
Score ≥ 5	102 (8.1%)	126 (8.1%)	>0.9
Score ≥ 9	24 (1.9%)	33 (2.1%)	0.8

COVID-19, coronavirus disease 2019; SOTR, solid organ transplant recipient.

## DISCUSSION

In this national survey of 1308 SOTRs, we found that only 51% intend to obtain a SARS-CoV-2 vaccine once available. We identified concerns about side effects, incompatibility with personal health conditions, lack of rigor in testing and development, and low efficacy as major barriers. Our study highlights the large amounts of skepticism toward a potential vaccine. These findings are striking in the face of available preliminary reports at the time of survey administration of high efficacy in phase III clinical trials, specifically 90% and 94.5% efficacy in the SARS-CoV-2 vaccines BNT162b1 and mRNA-1273, respectively.^2,22^ It seems that skepticism arising from the speed with which vaccines are being developed and tested may offset early acceptance. Reassuringly, willingness to be vaccinated was close to 90% among SOTRs if a recommendation came from a transplant provider, foreshadowing the critical role transplant providers may play.

The average vaccine, taken from the preclinical phase, requires a development timeline of approximately 11 y.^23^ Even after the Ebola outbreak of 2014, the accelerated development of the first Ebola vaccine took 5 y.^24^ The COVID-19 pandemic has shifted the development paradigm from a linear sequence to a parallel sequence, with multiple stages being executed simultaneously. It has taken only 11 mo from the time Chinese authorities shared the genetic sequence of the novel coronavirus to the first injections of a vaccine to the public.^25^ However, the emphasis on speed has provoked public anxiety. Our results confirm previous findings of quality control fears and concern for the fast pace of development and testing.^13,26^ Despite the reassurance of unwavering regulatory safeguards by the FDA,^27^ it seems that months of mixed messages from various sources about COVID-19 may have eroded public trust. We found that the likelihood of accepting a potential vaccine did marginally increase after the efficacy announcement of the mRNA-1273 vaccine, but still far below conservatively projected herd immunity thresholds. Widespread vaccination among family members and household contacts of SOTRs may provide a level of protection even if herd immunity is not achieved. However, we found similar rates of intention to accept a vaccine between household contacts (57.6%) as compared to those who do not live with an SOTR (61.3%). This low rate of vaccine acceptance is alarming, especially given that only half (52.2%) of household contacts believe that SOTRs should receive a vaccine. The need for reassurance, education, and vaccine promotion will be all the more necessary in the upcoming days to weeks as vaccines become increasingly available to transplant recipients.

Hesitancy to incur any vaccine-related risks was demonstrated by the significant need to understand organ transplant compatibility and to know physician recommendations before considering a potential SARS-CoV-2 vaccine. However, this may also be one of the greatest areas to promote vaccine acceptance. Although half of SOTRs were skeptical about a potential vaccine, 86.8% would be willing to receive a vaccine should that recommendation come from a transplant provider, whereas only 64% would be willing if it came from a primary care physician. These results highlight the incredible amount of trust between the SOTR and their transplant providers, a bond that can be the defining influence in vaccine acceptance. A recommendation by a trusted transplant provider can alleviate, or at least reduce, many fears SOTRs may have toward a new vaccine. Transplant providers will likely have a crucial role to play in protecting this population from infection by promoting vaccine adherence.

However, such promotion may be limited because of social distancing and pandemic-related restrictions. Therefore, additional alternative methods to promote vaccine adherence among transplant recipients should be considered. Recently, the *Am J Transplant* (AST) issued a statement recommending COVID-19 vaccination for most transplant recipients and candidates.^28^ Further adoption statements by other transplant societies could act as a rapid, widespread, and unified message to the transplant community, quelling suspicion and fear. The rise of online clinical visits and virtual meetings can also be leveraged to reach large audiences, whereas virtual town halls and community dialogues can act as a gateway toward the dissemination of vaccine information and physician recommendations. Our study found that even a recommendation from a nonphysician member of the transplant team was able to convince 72% of kidney transplant recipients to receive a vaccine. Nurse or coordinator-led group education sessions, transplant program position statements to patients, and other institutional outreach may all serve as the foundation to widespread vaccine acceptance in transplant recipients.

Those who were hesitant to receive a vaccine were more likely to be Black, of lower household income, and lower educational attainment. These findings are consistent with other studies that have found that lower socioeconomic status was negatively associated with vaccination rates,^29–32^ and the existence of wide racial disparities in vaccination.^33–35^ For example, one study of adult influenza vaccination demonstrated that African Americans had significantly lower odds of receiving an influenza vaccine compared to Whites (odds ratio = 0.55, 95% confidence interval, 0.42-0.72). These socioeconomic and racial disparities are of even higher concern given the increased rates of COVID-19 infection and mortality in disadvantaged populations,^36,37^ and among African American communities.^38,39^ Our findings further emphasize the importance of outreach to these vulnerable populations in which increased impact, coupled with lower vaccination rates, may further amplify health disparities.

This study is the first national survey to investigate the attitudes of SOTRs toward a potential SARS-CoV-2 vaccine. Some of the strengths of this study include a wide geographical distribution of responses and large sample size. We were not only able to collect attitudes about a potential vaccine but also identify the specific factors influencing vaccine hesitancy. Furthermore, the timing of our survey administration was during the peak of vaccination interest as large pharmaceutical companies began publishing reports of efficacy. We were able to distinguish whether certain announcements changed vaccine attitudes, making these findings particularly pertinent. Our study provides a framework for transplant providers to target education and anticipatory guidance efforts for SOTRs regarding these novel vaccines and, ideally, reduce vaccine hesitancy.

However, there are some limitations. Study respondents were largely White and with college degrees. The generalizability of this study to other populations may be limited. However, we do believe that the significant hesitancy captured in this study is likely on the conservative side, given that vaccine uptake is less among non-White, socially disadvantaged groups as described above. Additionally, our overall response rate was low, which may lead to participation bias. However, our response rate was consistent with similar large-scale surveys and our SOTR-specific response rate was quite high.^11,16^ Furthermore, we recognize that survey distribution through the NKF’s network may reflect engaged patients and families, and thus may not be generalizable to the entire population of kidney transplant recipients. This cross-sectional survey was also conducted in a rapidly changing landscape in which vaccine information is quickly evolving. Since the distribution of this survey, complete reports of the BNT162b1 vaccine (Pfizer Inc.) and mRNA-1273 vaccine (Moderna Inc.) have been published, emergency use authorizations have been granted, and millions of vaccines have already been distributed. The AST has also issued a statement recommending COVID-19 vaccination for most transplant recipients and candidates.^28^ Although individual attitudes may have shifted, we believe that the importance of transplant providers in providing reassurance and guidance to their patients likely remains true. Lastly, there may be potential for nonresponse bias as this study was administered via email and social media platforms, a form of communication that may not be used by all.

In conclusion, this national survey of SOTRs demonstrated heterogeneity in vaccine attitudes amongst SOTRs. Concerns about side effects, incompatibility with conditions, and lack of rigor in testing and development were identified as major barriers towards vaccine acceptance. Additional data on safety in the SOTR population may be needed to further convince those with hesitancy to pursue vaccination. Nonetheless, advocacy by transplant providers may lead to maximal rates of vaccination among SOTRs. Future research is needed to better understand vaccine behaviors in SOTRs and may promote vaccine acceptance.
